# The Impact of Gender-Affirming Hormone Therapy on Seizure Occurrence in Transgender and Gender-Diverse Individuals

**DOI:** 10.3390/jcm14103550

**Published:** 2025-05-19

**Authors:** Camille Blackman, Diane Saab, Danielle Mayorga-Young, Danielle Sim, Fan Liang, Emily L. Johnson, Bashar A. Hassan

**Affiliations:** 1 Department of Plastic and Reconstructive Surgery, Center for Transgender and Gender Expansive Health, Johns Hopkins Hospital, Baltimore, MD 21287, USA; cblackm8@jh.edu (C.B.); dsaab1@jh.edu (D.S.); danielle_mayorgayoung@urmc.rochester.edu (D.M.-Y.); dsim2@jh.edu (D.S.);; 2 University of Illinois College of Medicine, Chicago, IL 60612, USA; 3 University of Texas Southwestern Medical School, Dallas, TX 75390, USA; 4 Department of Neurology, Johns Hopkins University School of Medicine, Baltimore, MD 21205, USA

**Keywords:** epilepsy, seizure disorder, transgender, gender diverse, hormone therapy

## Abstract

**Background/Objectives**: Gender-affirming hormone therapy (GAHT) is an essential component of care for transgender and gender-diverse (TGD) individuals, yet its impact on seizure occurrence remains unclear. Given the known influence of hormonal fluctuations on seizure activity, this study evaluates whether GAHT affects seizure frequency in TGD individuals with a history of seizures. **Methods**: We conducted a retrospective cohort study of TGD individuals with a documented history of seizures who initiated GAHT between January 2002 and November 2024. Patients with inadequate follow-up, poor anti-seizure medication adherence, or concurrent feminizing GAHT (FHT) and masculinizing GAHT (MHT) use were excluded. The primary outcome was seizure occurrence before and after GAHT, subdivided into FHT vs. MHT. **Results**: Of 4391 TGD individuals, 34 met the inclusion criteria. Among 28 patients who had seizures before GAHT, 10 (35.7%) continued to have seizures after, while 18 (64.3%) did not. Seizure occurrence significantly decreased after GAHT: the proportion of individuals who experienced seizures before but not after GAHT was significantly greater than the proportion of individuals who experienced seizures after but not before GAHT (18/34, 52.9%; 6/34, 17.6%; *p* = 0.025). Among 21 patients on MHT, the proportion of patients who experienced seizures before but not after MHT was greater than the proportion of patients who experienced seizures after but not before MHT, but the difference was not statistically significant (11/21, 52.4%; 3/21, 14.3%; *p* = 0.06). FHT had no significant impact on seizure occurrence. **Conclusions**: GAHT was not associated with increased seizure occurrence in this small study. New-onset seizures occurred equally in the FHT and MHT groups, suggesting no disproportionate effect of estrogen-containing regimens. Our results suggest that GAHT might be safe in TGD individuals with epilepsy, though those with poorly controlled seizures may require closer monitoring. Further research may clarify the impact of GAHT on seizure disorders.

## 1. Introduction

Gender-affirming hormone therapy (GAHT) is a key component in the medical care of many transgender and gender-diverse (TGD) individuals seeking to alleviate their gender dysphoria or prepare for gender-affirming surgery (GAS). Feminizing hormone therapy (FHT), including estradiol, synthetic progestins, and androgen blockers, and masculinizing hormone therapy (MHT), such as testosterone, are often utilized depending on the patient’s gender-affirming goals [1,2]. While GAHT is generally considered safe and effective in achieving the desired physical and psychosocial outcomes [3], the influence of these hormonal regimens on comorbid conditions, particularly neurological disorders such as epilepsy, remains underexplored.

Epilepsy affects approximately 1.2% of the United States population [4], and emerging research indicates that individuals from sexual and gender minority groups may have a higher prevalence of active epilepsy compared with the general population [5]. Although the association between sex hormones and seizure activity is complex, most existing studies have focused on cisgender populations.

Studies in cisgender women suggest that estrogen may have proconvulsant effects, while progesterone may have anticonvulsant effects through the modulation of GABAergic activity [2,6]. As FHT regimens often combine estrogen, synthetic progesterone, and/or spironolactone, with adjustments made based on individual comorbidities [7], the overall impact of FHT on seizure control in TGD individuals may be hard to predict, warranting further investigation. Similarly, testosterone has been reported to exert both pro- and anticonvulsant effects [8,9,10], though some studies suggest that its primary effect is anticonvulsant [11,12]. While these anticonvulsant properties are documented in cisgender individuals, data on the impact of testosterone on seizure occurrence in TGD individuals undergoing GAHT remain limited [11].

GAHT can also interact with certain anti-seizure medications (ASMs). Estradiol may worsen seizure control by lowering serum lamotrigine concentrations by inducing its metabolism [9], while enzyme-inducing ASMs reduce circulating levels of estrogen and testosterone, potentially diminishing the efficacy of GAHT [13]. Because some GAHT drugs may affect the seizure threshold, TGD individuals on GAHT might face a higher risk of seizures. To explore these concerns, we examine two key questions: (1) Is GAHT associated with a greater seizure frequency in TGD individuals? (2) Does the type of GAHT—whether feminizing or masculinizing—influence the occurrence of seizures after therapy? Given prior evidence that estrogen may lower the seizure threshold and testosterone may be an overall anticonvulsant, we hypothesized that estrogen-containing GAHT regimens would increase seizure occurrence while testosterone-containing regimens would have a protective effect. We aim for our findings to serve as an initial step in elucidating the relationship between GAHT and seizure disorders, ultimately guiding clinical decision-making and best practices for this population.

## 2. Materials and Methods

### 2.1. Population

We conducted a pilot retrospective cohort study of TGD individuals with a documented history of seizures at a single institution, the Johns Hopkins Hospital, covering the period from January 2002 to November 2024. We used the STROBE cohort checklist when writing our report.

The patients we initially included for chart screening were those with a history of seizures, gender dysphoria, and GAHT use. For a history of seizures, we used the ICD codes G40, R56.1, and R56.9 to include as many patients with seizures as possible; we later included only the patients with reported or confirmed epileptic seizures via chart abstraction. Gender dysphoria and GAHT use were determined by physician and chart documentation. The exclusion criteria were inadequate clinical documentation defined as insufficient information in the patient’s electronic medical record to determine the patient’s seizure profile, ASM use, and/or GAHT regimen at any of the relevant timepoints in this study: before or after starting GAHT. The other exclusion criteria included patients adhering to <70% of their ASM regimen; those with significant hepatic, renal, or hematological abnormalities; use of investigational drugs or devices within 30 days prior to GAHT initiation; patients taking medications from both MHT and FHT regimens simultaneously; and patients whose seizures occurred only in the setting of known provocations, such as infantile febrile seizures, substance or medication use, alcohol withdrawal, or solely functional seizures.

Patients were included in the initial study cohort only if their seizures were either reported as epileptic after receiving care at an outside institution or confirmed through clinical documentation to be epileptic in nature. For these patients, we collected patient demographics, comorbid conditions, details of GAHT regimens, and seizure characteristics. Epilepsy was designated as “confirmed” if the epileptic activity was recorded by an electroencephalogram (EEG) or if a neurologist at our institution documented the patient’s seizures as epileptic in a note or as a diagnosis. The seizures were further classified as “provoked” if a physician documented in the medical record that they were triggered by specific factors, including medication nonadherence, sleep deprivation, new medications, estrogen exposure in patients on lamotrigine, alcohol or substance use, significant stress, or head trauma. The follow-up period was considered to be the duration between the first clinical visit before starting GAHT to the most recent clinical visit after starting GAHT.

### 2.2. Exposure

The main exposure of the study was the use of GAHT. We classified GAHT into any form of MHT (testosterone) or FHT (estrogen, progesterone, medroxyprogesterone, spironolactone, or combinations of these medications).

### 2.3. Outcome

The main objective was to evaluate whether GAHT—either feminizing or masculinizing—was associated with seizure activity by comparing seizure occurrence before and after therapy. The primary outcome of the study was any seizure occurrence, treated as a binary outcome (yes/no).

Seizure activity prior to GAHT was defined as any seizures documented by a neurologist, on patient history, in emergency medical records, or found in the patient’s chart, at any point before starting GAHT. Seizure activity on GAHT was defined as any seizures as documented by a neurologist, on patient history, in emergency medical records, or found in the patient’s chart, at any point after starting GAHT.

The secondary purpose of the study was to assess the potential association between the type of GAHT and seizure activity in patients with a history of seizures and confirmed epilepsy.

### 2.4. Statistical Analysis

We performed descriptive statistics on the overall study population. To assess the association between GAHT and seizures, we compared seizure activity before versus after initiating GAHT during the complete study period using McNemar’s test. Continuity correction was applied to adjust for small sample size biases. The same analysis was repeated after stratifying by FHT and MHT to assess the impact of each on seizure activity. To control for the effect of ASMs on seizure occurrence, the same analysis was stratified by whether ASM dosing was increased or unchanged after GAHT.

We performed two sensitivity analyses comparing FHT versus MHT. We included patients with documented seizures prior to starting GAHT in the first analysis and focused exclusively on patients with a confirmed diagnosis of epilepsy in the second analysis.

We used Fisher’s exact test and Chi-square test to compare the proportions of seizure occurrence in TGD individuals initiating FHT versus MHT. All the statistical analysis was performed using the R 4.4.1 statistical software (R Foundation for Statistical Computing, Vienna, Austria). A *p*-value < 0.05 was considered significant.

## 3. Results

### 3.1. Demographic Characteristics

Of 4391 TGD individuals seen at the Johns Hopkins Hospital, 86 (1.9%) had a documented history of seizures at any time (before or after starting GAHT), and 34 (0.8%) met the inclusion criteria and were analyzed. All 34 of these individuals have reported epilepsy or had confirmed epilepsy. Table 1 shows the demographics, comorbidities, gender-affirming care characteristics, and seizure characteristics of the study population.

Of the 34 included individuals, 12 (35.3%) were assigned male at birth (AMAB), while 22 (64.7%) were assigned female at birth (AFAB). In terms of gender identity, around half (18, 53.0%) identified as transmen, while 10 (29.4%) identified as transwomen. The majority of our study population was White (20, 58.8%) and had medical (30, 88.2%) and psychiatric (27, 79.4%) comorbidities. Insurance types were as follows: private for 15 (44.1%) patients, Medicare for five (14.7%), and Medicaid for 14 (41.2%) (Table 1).

### 3.2. Gender-Affirming Care

The average (standard deviation [SD]) age at GAHT initiation was 28 (12) years. The majority of our study population underwent MHT (21, 61.8%), while the rest underwent FHT (13, 38.2%). The median (interquartile [IQR]) duration of GAHT across both groups was 51 (31–84) months and the median (IQR) follow-up duration was 4 (3–7) years.

Of 21 patients who underwent MHT, 17/21 (81.0%) used testosterone cypionate injections, 3/21 (14.3%) used testosterone enanthate injections, and 1/21 (4.8%) did not report testosterone formulation. Injections were either intramuscular, subcutaneous, or unspecified.

Of 13 patients who underwent FHT, 5/13 (38.5%) used estradiol valerate 20 mg/mL injections. Oral estradiol pills were used by 7/13 (53.8%) patients. The regimens included estradiol as the sole drug; estradiol coupled with a progesterone, medroxyprogesterone injections or pills, or progesterone pills; estradiol coupled with spironolactone pills; and estradiol coupled with both progesterone and spironolactone. One patient was taking spironolactone for its anti-androgenic effects without any progesterone or estradiol. Information on the specific GAHT dose and regimen of each patient is detailed in Supplemental Table S1.

Half of the study population (17, 50.0%) underwent GAS, most commonly chest masculinization surgery (12, 35.3%) (Table 1).

### 3.3. Seizure Characteristics

The mean (SD) age at seizure onset was 15 (11) years, whereas the mean (SD) age for a patient’s initial neurology appointment prior to GAHT was 25 (13) years. A total of 7 (20.6%) patients reported a family history of epilepsy, 21 (61.8%) patients reported no family history of epilepsy, and 6 (17.6%) patients had an unknown family history of epilepsy (Table 1).

Among 34 patients, definite epilepsy was confirmed in 18 (52.9%) patients via EEG or the documentation of an epilepsy diagnosis by a neurologist. The remaining 16 patients (47.1%) had only reported seizures without confirmatory diagnostic evidence. EEG was performed in 21 (61.8%) patients. Among the 20 patients whose EEGs were reported, the findings were as follows: 12/20 (57.1%) patients had a normal EEG, 2/20 (9.5%) exhibited generalized epileptiform changes, 5/20 (23.8%) showed focal epileptiform changes, and 1/20 (4.8%) patient demonstrated non-epileptiform abnormalities. Eleven patients’ seizures were unknown whether they were epileptic or non-epileptic.

Neuroimaging was performed in 22 patients: CT only in 8/22 patients (36.4%), and CT and MRI in 14/22 (63.6%) patients. Lesional abnormalities were detected in 5/22 (22.7%) (Table 1).

Eighteen (52.9%) patients used ASM within 6–12 months before starting GAHT, while sixteen (47.1%) did not (Table 1). Twenty (60%) patients used ASM in the 6–12-month period after GAHT, while fourteen (41.2%) did not (Table 1). Twenty-four patients had stable ASM regimens, while ten required an increase in their ASM.

### 3.4. GAHT and Seizure Activity

The median (IQR) follow-up duration was 4 (3–7) years.

Twenty-eight (82.4%) patients experienced seizures before GAHT. Of these 28, 10/28 (35.7%) continued to experience seizures after GAHT, while 18/28 (64.3%) did not. The proportion of patients who experienced seizures before but not after GAHT was significantly greater than the proportion of patients who experienced seizures after but not before GAHT (18/34, 52.9%; 6/34, 17.6%; *x*^2^ = 6, *p* = 0.014 before continuity correction; *x*^2^ = 5, *p* = 0.025 after continuity correction; Table 2). Of the six patients who had no seizures before GAHT but developed seizures afterward, three were on FHT and three were on MHT (Table 3).

Seizure occurrence decreased after MHT, but the result only approached significance. Among the 21 patients on MHT, the proportion of patients who experienced seizures before but not after MHT was greater than the proportion of patients who experienced seizures after but not before MHT (11/21, 52.4%; 3/21, 14.3%; *x*^2^ = 4.57, *p* = 0.03 before continuity correction, *x*^2^ = 3.50, *p* = 0.06 after continuity correction; Table 3).

Among the 13 patients on FHT, there was no significant change in the occurrence of seizures before versus after FHT (*x*^2^ = 0.9, *p* = 0.34 after continuity correction; Table 3).

### 3.5. GAHT and Seizure Activity in Patients with Prior History of Seizures or Confirmed Epilepsy

The demographic characteristics of the 28 patients with seizures prior to GAHT are displayed in Table 4. There was no significant difference in post-GAHT seizure occurrence among those on FHT versus MHT (3/10, 30.0%; 7/18, 38.9%; *p* = 0.7; Table 5).

Among the 15 TGD individuals with definite, confirmed epilepsy prior to GAHT as defined by epileptiform EEG or expert physician documentation, seizure occurrence was also not significantly different among those on FHT versus MHT (2/4, 50.0%; 6/11, 54.5%; *p* = 1.0; Table 6).

## 4. Discussion

This study aimed to determine whether GAHT—either feminizing or masculinizing—affects seizure occurrence in TGD individuals, and whether the type of GAHT (FHT vs. MHT) influences post-therapy seizure occurrence in those with a history of seizures or confirmed epilepsy. Based on prior evidence that estrogen may lower the seizure threshold and testosterone may have anticonvulsant effects, we hypothesized that estrogen-containing GAHT regimens would increase seizure occurrence while testosterone-containing regimens would have a protective effect. Our analysis focused on seizure activity as a binary outcome (yes/no), allowing us to assess the overall changes in seizure occurrence and explore the potential differences between the FHT and MHT regimens.

Our results suggest that GAHT does not increase the risk of seizure occurrence. Moreover, among the TGD individuals with a pre-GAHT seizure history or confirmed epilepsy, seizure occurrence after GAHT did not significantly differ between the FHT and MHT groups, suggesting that one therapy type does not influence seizure occurrence more than the other in patients predisposed to seizures. While the small sample size limits definitive conclusions, these findings are reassuring as GAHT does not appear to be associated with a higher incidence of new-onset seizures, along with no difference between the FHT and MHT regimens.

Prior research on sex hormones and seizure activity in cisgender individuals highlights the complex interactions between hormone levels and neuronal excitability. Estradiol has been associated with increased seizure susceptibility, likely due to its role in stimulating excitatory glutamate activity while simultaneously reducing inhibitory gamma-aminobutyric acid (GABA) transmission [14]. In contrast, progesterone and its neuroactive metabolite, allopregnanolone, exert anticonvulsant effects by enhancing GABAergic inhibition [6,7]. However, synthetic progestins commonly found in oral contraceptives lack these neuroprotective properties, with the exception of medroxyprogesterone [6]. The lack of an increase in seizure occurrence in this small study among those on FHT suggests that either the doses used in GAHT were insufficient to provoke seizures in most individuals, or that ASMs and other physiological factors mitigated the proconvulsant effects of estradiol.

Testosterone, on the other hand, appears to exert anticonvulsant effects, but its impact on seizure modulation depends on its metabolic pathway. Testosterone can be converted to estradiol via aromatization, potentially increasing seizure susceptibility, or it can be reduced to dihydrotestosterone (DHT) and further metabolized into 3α-androstanediol (3α-Diol), a neurosteroid with GABA-A receptor-modulating anticonvulsant properties [15]. Studies in cisgender men suggest that testosterone and its metabolites enhance GABAergic inhibition, reducing neuronal excitability [11,12]. Animal models similarly demonstrate that testosterone-derived neurosteroids can exert protective effects against seizures by modulating neuronal firing and increasing seizure thresholds [16]. While we did not observe a significant decrease in seizure occurrence after MHT in this study after correcting for a small sample size, there was a trend toward reduced seizure occurrence in those on MHT. The reduction is consistent with prior findings and suggests that testosterone-based regimens are at least not harmful in seizure-prone individuals.

Neuroinflammation in epilepsy may also contribute to the observed outcomes. Testosterone has been shown to possess anti-inflammatory properties that may mitigate seizure activity by reducing cytokine-mediated neuronal excitability [17]. Conversely, estrogen has varying effects on pro-inflammatory pathways that could exacerbate seizure activity in susceptible individuals [18]. These immunomodulatory properties of GAHT warrant further investigation, particularly in the context of epilepsy pathophysiology.

Hormonal interactions with ASMs further influence seizure risk in TGD individuals receiving GAHT. Estradiol accelerates the metabolism of lamotrigine, leading to lower serum drug concentrations [19] and potential seizure breakthroughs in individuals on this ASM. In contrast, enzyme-inducing ASMs such as phenytoin, carbamazepine, and phenobarbital can reduce the circulating levels of estradiol and testosterone [20], potentially diminishing the efficacy of GAHT. In this small cohort, we did not find differences in seizure control among those on estrogen-containing FHT and those on MHT.

The clinical implications of our findings highlight the importance of individualized treatment approaches when managing epilepsy in TGD individuals on GAHT. While GAHT does not appear to be associated with an increased seizure risk in this small study, patients with poorly controlled epilepsy still require closer monitoring, particularly individuals on estradiol-containing regimens and lamotrigine.

Clinicians should be aware of potential GAHT-ASM interactions and consider adjusting the medication dosages accordingly to prevent seizure breakthroughs or hormonal insufficiency when needed. Future research should explore seizure frequency in more detail and whether modifying GAHT regimens based on seizure risk could further optimize neurological outcomes in this population.

### Limitations

Several factors limit the interpretation of this study’s findings. First, the small sample size (*n* = 13 for FHT; *n* = 21 for MHT) limits statistical power and generalizability; thus, we may have been unable to detect meaningful differences despite our use of continuity correction. Second, we did not stratify by hormone dosage, therapy duration, non-compliance, or treatment frequency, which weakens the inferential power of our findings in terms of the mechanistic understanding of GAHT and seizure control. Including these variables would have required further subgrouping, leading to an even smaller effective sample size and possibly inflating the risk of a Type II error. Third, we did not account for the potential effects of hormone-altering GAS, such as orchiectomy or oophorectomy. These procedures can alter endogenous hormone levels and may affect seizure thresholds. Fourth, the focus of our study on patients who underwent GAHT did not allow us to compare individuals who underwent versus those who did not undergo GAHT. Importantly, due to the nature of the retrospective chart review (and the fact that many patients do not receive gender-affirming care and neurology care at the same institution), we were unable to ascertain exact seizure frequency data or additional characteristics of epilepsy for some patients, which precludes us from drawing conclusions about reduction or increase in seizure frequency other than presence or absence of seizures. The differing length of time each patient had records available before and after GAHT also limited the ability to directly compare seizure frequency. Our results are correlational and suggest possible relationships rather than establish causation. Nevertheless, in this understudied population, our study represents an initial step in studying the relationship between GAHT and seizures and provides reassurance for initiating GAHT in patients with epilepsy.

Future studies should address these limitations by enrolling larger cohorts and incorporating additional stratifications to clarify how GAHT, surgical interventions, and epilepsy management intersect.

## 5. Conclusions

These findings suggest that GAHT—whether feminizing or masculinizing—may not be associated with increased seizure occurrence in TGD individuals. However, individuals with poorly controlled or refractory epilepsy always require close monitoring and collaboration with neurology to optimize care. Our work highlights the need for larger, prospective studies to clarify how hormone dosage, therapy duration, surgical interventions, and epilepsy management intersect in a TGD population. Such research will guide future best practices and ensure safer, more effective care for TGD patients with co-existing epilepsy.

## Figures and Tables

**Table 1:** Demographics, comorbidities, and seizure characteristics of our patient population.

Characteristics of the Study Patient Population		
Sample Size, *n* = 34		*n* (%)
**Demographics**		
Sex assigned at birth	Female	22 (64.7)
	Male	12 (35.3)
Gender identity	AFAB, transmen	18 (53.0)
	AMAB, transwomen	10 (29.4)
	AMAB, non-binary	2 (5.9)
	AFAB, non-binary	2 (5.9)
	AFAB, women	2 (5.9)
Race and ethnicity	White, non-Hispanic	20 (58.8)
	Black, non-Hispanic	12 (35.3)
	Asian, non-Hispanic	1 (2.9)
	American Indian or Alaska native, non-Hispanic	1 (2.9)
Employment status	Unemployed	18 (52.9)
	Employed	14 (41.2)
	Full-time student	2 (5.8)
Insurance type	Private	15 (44.1)
	Medicaid	14 (41.2)
	Medicare	5 (14.7)
**Medical Comorbidities**		
Presence of any medical comorbidity		30 (88.2)
Obesity		15 (44.1)
Migraine		12 (35.3)
Chronic pain disorder		5 (14.7)
Stroke		1 (2.9)
Neoplasia (Tuberous Sclerosis)		1 (2.9)
**Psychiatric Comorbidities**		
Presence of any psychiatric comorbidity		27 (79.4)
Depression		23 (67.6)
Anxiety		19 (55.9)
ADHD		10 (29.4)
Other		15 (44.1)
**Gender-Affirming Care Characteristics**		
Age at start of GAHT, mean ± SD, years		28 ± 12
Type of GAHT	MHT	21 (61.8)
	FHT	13 (38.2)
Any type of GAS		17 (50.0)
Type of GAS	Masculinizing chest surgery	12 (35.3)
	Both masculinizing chest and genital surgery	2 (5.9)
	Feminizing chest surgery	1 (2.9)
	Feminizing genital surgery	1 (2.9)
	Facial feminization surgery	1 (2.9)
	Not applicable	17 (50.0)
**Seizures Characteristics**		
Age of seizure onset, mean ± SD, years		15 ± 11
Age at first neurology appointment closest to starting GAHT, mean ± SD, years *(available for n = 19)*		25 ± 13
Family history of seizures (*available for n = 28)*		7 (20.6)
Epilepsy confirmed by EEG or expert physician assessment		18 (52.9)
EEG performed		21 (61.8)
EEG findings (*available for n = 20)*	Normal	12 (57.1)
	Focal epileptiform	5 (23.8)
	Generalized epileptiform	2 (9.5)
	Non-epileptiform abnormality	1 (4.8)
Neuroimaging performed	MRI with or without CT	13 (38.2)
	CT only	8 (23.5)
Neuroimaging findings (*available for n = 21)*	Non-lesional (normal)	16 (76.2)
	Lesional	5 (23.8)
Use of anti-seizure medications in 6–12 months before GAHT		18 (52.9)
Use of anti-seizure medications in 6–12 months after GAHT		20 (58.8)
Seizures before GAHT		28 (82.4)
Seizures after GAHT		16 (47.1)
Type of seizure in 12 months after starting GAHT *(available for n = 5)*	Unprovoked	3 (18.8)
	Provoked	2 (12.5)

ADHD: attention-deficit hyperactivity disorder; AFAB: assigned female at birth; AMAB: assigned male at birth; CT: computed tomography; EEG: electroencephalogram; FHT: feminizing hormone therapy; GAHT: gender-affirming hormone therapy; GAS: gender-affirming surgery; MHT: masculinizing hormone therapy; MRI: magnetic resonance imaging; SD: standard deviation.

**Table 2:** Contingency tables and McNemar test results comparing seizure occurrence before and after gender-affirming hormone therapy (*n* = 34).

Gender-Affirming Hormone Therapy (GAHT), *n* = 34 *n* (%)	After GAHT: Seizures	After GAHT: No Seizures	*p*-Value without Continuity Correction	*p*-Value with Continuity Correction
Before GAHT: Seizures	10 (29.4)	18 (52.9)	0.014 *	0.025 *
Before GAHT: No Seizures	6 (17.6)	0 (0.0) **		

* = *p*-value < 0.05; ** patients without seizures before or after GAHT were not included based on the inclusion and exclusion criteria; the “no seizures” concordant pair count does not affect the McNemar *p*-value.

**Table 3:** Contingency tables and McNemar test results comparing seizure occurrence before and after masculinizing (*n* = 21) and feminizing (*n* = 13) hormone therapy.

Masculinizing Hormone Therapy, *n* = 21 *n* (%)	After: Seizures	After: No Seizures	*p*-Value Without Continuity Correction	*p*-Value with Continuity Correction
Before: Seizures	7 (33.3)	11 (52.4)	0.03 *	0.06
Before: No Seizures	3 (14.3)	0 (0.0) **		
**Feminizing Hormone Therapy, *n* = 13** ***n* (%)**	**After: Seizures**	**After: No Seizures**	***p*-Value Without Continuity Correction**	***p*-Value with Continuity Correction**
Before: Seizures	3 (23.1)	7 (53.8)	0.21	0.34
Before: No Seizures	3 (23.1)	0 (0.0) **		

* = *p*-value < 0.05; ** patients without seizures before or after GAHT were not included based on the inclusion and exclusion criteria; the “no seizures” concordant pair count does not affect the McNemar *p*-value.

**Table 4:** Characteristics of patients with pre-existing seizures before gender-affirming therapy hormone therapy (*n* = 28).

Characteristics of the Subgroup with Pre-Existing Seizures Before GAHT				
Sample Size, *n* = 28 *n* (%)		No Seizures After GAHT (*n* = 18, 64.3%)	Seizures After GAHT (*n* = 10, 35.7%)	*p*-Value
**Demographics**				
Sex assigned at birth	Male	6 (21.4)	4 (14.3)	1.0
	Female	12 (42.9)	6 (21.4)	
Gender identity	AFAB, trans male	10 (35.7)	4 (14.3)	–
	AMAB, trans female	5 (17.9)	3 (10.7)	
	AMAB, non-binary	1 (3.6)	1 (3.6)	
	AFAB, non-binary	1 (3.6)	2 (7.1)	
	AFAB, female	1 (3.6)	0 (0)	
Race and Ethnicity	White, non-Hispanic	10 (35.7)	6 (21.4)	0.8
	Black, non-Hispanic	7 (25.0)	4 (14.3)	
	Asian, non-Hispanic	1 (3.6)	0 (0)	
Employment status	Unemployed	10 (35.7)	6 (21.4)	0.3
	Employed	7 (25.0)	3 (10.7)	
	Full-time student	1 (3.6)	1 (3.6)	
Insurance type	Private	9 (32.1)	2 (7.1)	1.0
	Medicare	2 (7.1)	2 (7.1)	
	Medicaid	7 (25.0)	6 (21.4)	
**Medical Comorbidities**				
Medical Comorbidities		16 (57.1)	8 (28.6)	0.6
Obesity		9 (32.1)	2 (7.14)	0.2
Migraine		5 (17.9)	4 (14.3)	0.7
Chronic pain disorder		2 (7.1)	2 (7.1)	0.6
Neoplasia		1 (3.6)	0 (0.0)	1.0
Stroke		0 (0.0)	1 (3.6)	0.4
**Psychiatric Comorbidities**				
Psychiatric comorbidities		13 (46.4)	8 (28.6)	1.0
Depression		11 (39.3)	7 (25.0)	0.7
Anxiety		9 (32.1)	7 (25)	0.4
ADHD		5 (17.9)	2 (7.14)	1.0
Other		5 (17.9)	5 (17.9)	0.5
**Gender-Affirming Care**				
Age at start of GAHT (*years*), mean (SD)		29 (12.7)	11 (11.1)	0.7
Any type of GAS		7 (25.0)	6 (21.4)	0.4
Type of GAS	Masculinizing chest surgery	6 (21.4)	5 (17.6)	–
	Feminizing chest surgery	1 (3.5)	0 (0.0)	
	Both masculinizing chest and bottom surgery	0 (0.0)	1 (3.5)	
	Not applicable	11 (39.3)	4 (14.3)	
**Seizures Characteristics**				
Family history of seizures *(available for n = 23)*		5 (21.7)	1 (4.3)	0.7
Epilepsy confirmed		7 (25.0)	8 (32.0)	0.037 *
EEG findings, *(available for n = 16)*	Normal	3 (10.3)	5 (17.2)	–
	Focal epileptiform	4 (13.8)	0 (0.0)	
	Generalized epileptiform	1 (3.4)	0 (0.0)	
	Non-epileptiform abnormality	0 (0.0)	1 (3.4)	
Neuroimaging performed	None	9 (32.1)	4 (14.3)	0.9
	CT only	5 (17.9)	3 (10.7)	
	MRI	4 (14.3)	3 (10.7)	
Neuroimaging findings *(available for n = 16)*	Non-lesional	7 (46.7)	4 (26.7)	1.0
	Lesional	2 (13.3)	2 (13.3)	
Type of seizure *(available for n = 8)*	Unprovoked	3 (10.7)	3 (10.7)	–
	Mixed (provoked and unprovoked)	0 (0.0)	2 (7.1)	
Use of anti-seizure medications in 6–12 mo after GAHT		11 (39.3)	8 (28.6)	0.5
Anti-seizure medication dosage increase		4 (14.3)	3 (10.7)	1.0

* = *p*-value < 0.05; ADHD: attention-deficit hyperactivity disorder; AFAB: assigned female at birth; AMAB: assigned male at birth; CT: computed tomography; EEG: electroencephalogram; GAS: gender-affirming surgery; GAHT: gender-affirming hormone therapy; MRI: magnetic resonance imaging; SD: standard deviation.

**Table 5:** Chi-square analysis of the association between feminizing and masculinizing hormone therapy and the occurrence of seizures after gender-affirming hormone therapy (GAHT) in patients with a history of seizures prior to GAHT (*n* = 28).

Type of Gender-Affirming Hormone Therapy (GAHT), *n =* 28	Feminizing Hormone Therapy (*n* = 10)	Masculinizing Hormone Therapy (*n* = 18)	*p*-Value
Seizures after GAHT	3 (30.0)	7 (38.9)	0.7
No seizures after GAHT	7 (70.0)	11 (61.1)	

**Table 6:** Chi-square analysis of the association between feminizing and masculinizing hormone therapy and the occurrence of seizures after gender-affirming hormone therapy (GAHT) in patients with confirmed epilepsy before GAHT (*n* = 15).

Type of Gender-Affirming Hormone Therapy (GAHT), *n =* 15	Feminizing Hormone Therapy (*n* = 4)	Masculinizing Hormone Therapy (*n* = 11)	*p*-Value
Seizures after GAHT	2 (50.0)	6 (54.5)	1.0
No seizures after GAHT	2 (50.0)	5 (45.5)	

## Data Availability

The raw data supporting the conclusions of this article will be made available by the authors upon request.

## Supplementary Materials

The following supporting information can be downloaded at: https://www.mdpi.com/article/10.3390/jcm14103550/s1, Table S1: Summary table of patients included in the study.
