# Supplementary material for: The Impact of Gender-Affirming Hormone Therapy on Seizure Occurrence in Transgender and Gender-Diverse Individuals
Source: J Clin Med. 2025 May 19;14(10):3550. doi: 10.3390/jcm14103550 (PMC12111937; doi:10.3390/jcm14103550)
Supplement: Supplementary file 1 [file jcm-14-03550-s001.zip › jcm-3571441-supplementary.pdf]

Supplementary Table S1. Summary table of patients included in the study.

Patient No.	Age of seizure onset (years)	Sex assigned at birth	Gender identity	GAHT type	GAHT dose	GAHT regimen	Duration of follow-up after starting GAHT (days)	Seizure before GAHT? Yes/No	Seizure after GAHT? Yes/No	Anti-seizure medication dose and regimen before starting GAHT	Change in ASM: increased/no change
1	10	AFAB	Man	MHT	Testosterone cypionate 60mg	60mg SQ 1x per week	1185	Yes	No	Carbamazepine 100 mg qAM, 200mg qhs	No change
2	41	AFAB	Man	MHT	Testosterone cypionate 20mg	200mg IM q14d	3823	No	Yes	None	Increased - Levetiracetam 1500 mg BID
3	7	AFAB	Woman	FHT	Medroxyprogesterone 150mg (for menstrual suppression)	150mg IM q3mo	959	Yes	No	None	No change
					Leuprolide 11.25 mg	11.25 mg IM q3mo					
4	19	AFAB	Non-binary	MHT	Testosterone cypionate 80mg	80mg IM weekly	1263	Yes	No	Carbamazepine 200 mg 2.5 times/day	Increased - Carbamazepine 300 mg BID
5	19	AFAB	Woman	FHT	Medroxyprogesterone 150mg (for menstrual suppression)	150mg IM q3mo	1744	No	Yes	None	No change
6	24	AFAB	Man	MHT	Testosterone cypionate 50mg	50mg SQ weekly	677	Yes	No	Clonazepam 0.5mg qhs, topiramate 100 mg qhs	No change
7	0	AFAB	Man	MHT	Testosterone cypionate 80mg	80mg SQ weekly	2895	Yes	No	Lamotrigine 100 mg BID, oxcarbazepine 300 mg BID, zonisamide 200 mg BID, lacosamide 300 mg BID	Increased - Lamotrigine 200 mg BID, oxcarbazepine 600 mg BID, zonisamide 100 mg qAM and 200 mg

											qhs, lacosamide 300 mg BID
8	NR	AMAB	Woman	FHT	Estradiol (ESTRACE) 2mg tablets	4 x2mg tablets (8mg total) PO daily	558	Yes	No	Lamotrigine 200mg, Levetiracetam 1250 mg BID	Increased - Lamotrigine 150mg BID, Levetiracetam 1500 mg BID
					Spironolactone (ALDACTONE) 25mg tablet	25mg BID					
9	25	AFAB	Man	MHT	Testosterone cypionate 80mg	80mg SQ weekly	2291	No	Yes	Carbamazepine 200 mg BID; lamotrigine 100mg BID	Increased - Lamotrigine 150 mg BID
10	0	AMAB	Woman	FHT	Estradiol (ESTRACE) 6mg	6mg daily	389	Yes	No	None	No change
					Spironolactone (ALDACTONE) 150mg	150mg qd					
11	13	AFAB	Man	MHT	Testosterone 100mg injection	100 mg IM every 2 weeks	1477	Yes	No	None	No change
12	20	AMAB	Woman	FHT	Estradiol valerate (DELESTROGEN) 20 mg/mL injection	20mg IM every 28 days	2216	Yes	No	Levetiracetam 750 mg BID	No change
					Medroxyprogesterone 5mg	5 mg qd					
					Spironolactone (ALDACTONE) 100mg tablet	100mg PO BID					
13	38	AFAB	Man	MHT	Testosterone	100mg/	1558	Yes	No	None	No change

					cypionate 100mg/mL	mL injection weekly					
14	7	AFAB	Man	MHT	Testosterone cypionate 40mg	40mg SQ weekly	799	Yes	No	Lamotrigine 175 mg BID, ethosuximide 250 mg qd	Increased - Lamotrigine 200 mg BID but no ethosuximide
15	11	AFAB	Man	MHT	Testosterone enanthate (Xyosted) 50mg	50mg SQ weekly	1470	Yes	No	Oxcarbazepine 600 mg AM, 900mg PM	No change
16	24	AMAB	Woman	FHT	Estradiol valerate (DELESTROGEN) 20mg/mL injection	20 mg/mL IM injection every 14 days	5000	No	Yes	None	No change
					Spironolactone 100mg	100mg qd					
17	1	AFAB	Man	MHT	Testosterone cypionate 200mg/mL	200mg/mL injection	768	Yes	No	Levetiracetam 500 mg BID, Topiramate 200 mg BID, lamotrigine 50 mg qd	No change
18	21	AFAB	Man	MHT	Testosterone cypionate 200mg/mL	25mg SQ weekly	2115	No	Yes	None	Increased - Gabapentin 300 mg TID
19	16	AMAB	Woman	FHT	Estradiol (ESTRACE) 1mg tablet	3 mg PO daily	1028	Yes	No	Lamotrigine 350 mg qd	No change
					Spironolactone (ALDACTONE) 25mg tablet	75 mg BID (3 tablets BID)					
20	14	AFAB	Man	MHT	Testosterone enanthate (DELATESTRYL) 200mg/mL	0.25mL (50mg total) SQ weekly	768	Yes	No	Topiramate 200 mg BID, divalproex 1750 mg qd	No change

21	5	AMAB	Non-binary	FHT	Estradiol valerate (DELESTROGEN) 20 mg/mL injection	6mg IM every 7 days	1280	Yes	No	None	No change
					Progesterone (PROMETRIUM) 200mg capsule	200 mg PO qd					
					Spironolactone 200 mg						
22	32	AMAB	Woman	FHT	Estradiol valerate 20 mg/mL oil	20mg IM	2255	No	Yes	None	No change
					Progesterone 200mg	200 mg PO qd in evening					
23	3	AMAB	Woman	FHT	Estradiol valerate (DELESTROGEN) 20 mg/mL injection		454	Yes	No	None	No change
					Progesterone (PROMETRIUM) 100mg capsule						
					Spironolactone (ALDACTONE) 50mg	50mg BID					
24	35	AFAB	Man	MHT	Testosterone cypionate 200 mg/mL injection	0.9mL (100 mg total) IM every 2 weeks	1150	Yes	No	Zonisamide 300 mg qd	No change
25	0	AMAB	Woman	FHT	Estradiol 2mg	2mg BID	1583	Yes	Yes	Lamotrigine 100mg qd (for	Increased - Lamotrigine

					Spironolactone 50mg	50mg BID				mood), lorazepam 0.5 bid (anxiety)	200mg qd, lacosamide 200mg
26	13	AMAB	Woman	FHT	Estradiol 2mg	2mg pill qd	5334	Yes	Yes	Divalproex 500mg qd and 1000mg qhs, topiramate 100mg bid	No
27	13	AFAB	Man	MHT	Testosterone enanthate (DELATESTRYL) 200MG/ML	200mg/mL IM oil weekly	2936	Yes	Yes	Levetiracetam 750mg bid	Increased - Levetiracetam 1500mg bid
28	<40	AMAB	Woman	FHT	Spironolactone (ALDACTONE) 100mg	100mg qd	2397	Yes	Yes	Divalproex 1000mg qd and 1500mg qhs, carbamazepine 400mg bid, carbatrol 100mg qd	No
29	12	AFAB	Non-binary	MHT	Testosterone cypionate 200mg/mL	200mg/mL injection, 18.75mg weekly	1210	Yes	Yes	Lacosamide 150mg bid	No
30	11	AFAB	Man	MHT	Testosterone cypionate 200mg/mL	20mg IM weekly	3663	Yes	Yes	NA	No
31	18	AFAB	Man	MHT	Testosterone cypionate 100mg/mL	100mg/mL injection every 14d	3456	Yes	Yes	NA	Levetiracetam 750mg bid
32	16	AFAB	Man	MHT	Testosterone cypionate 200mg/mL	50mg IM weekly	2919	Yes	Yes	Divalproex 250mg tid	No
33	20	AMAB	Non-binary	MHT	Testosterone cypionate 200mg/mL	30mg IM weekly	1841	Yes	Yes	Lamotrigine 100mg bid	No
34	2	AFAB	Man	MHT	Testosterone 45mg	45mg onto skin qd	894	Yes	Yes	NA	No

*Patients 3 and 5 de-transitioned after initially initiating GAHT.

ADHD: attention-deficit hyperactivity disorder
AFAB: assigned female at birth
AMAB: assigned male at birth
BID: bis in die, twice a day
EEG: electroencephalogram
FHT: feminizing hormone therapy
HT: hormone therapy
IM: intramuscular
MHT: masculinizing hormone therapy
PO: per os, orally
SQ: subcutaneous
TID: ter in die, thrice a day
QD: quaque die, once a day
